# The Use of Artificial Neural Networks and a General Discriminant Analysis for Predicting Culling Reasons in Holstein-Friesian Cows Based on First-Lactation Performance Records

**DOI:** 10.3390/ani11030721

**Published:** 2021-03-06

**Authors:** Krzysztof Adamczyk, Wilhelm Grzesiak, Daniel Zaborski

**Affiliations:** 1 Department of Animal Genetics, Breeding and Ethology, University of Agriculture in Krakow, al. Mickiewicza 24/28, 30-059 Kraków, Poland; 2 Department of Ruminants Science, West Pomeranian University of Technology, Klemensa Janickiego 29, 71-270 Szczecin, Poland; wilhelm.grzesiak@zut.edu.pl (W.G.); daniel.zaborski@zut.edu.pl (D.Z.)

**Keywords:** classification matrix, dairy cattle, data mining, gains chart, multilayer perceptron, sensitivity analysis

## Abstract

**Simple Summary:**

Routinely collected data on the performance of dairy cows are a valuable source of information on the beginning, course, and completion of their productive life. As a result, when using sufficiently accurate methods, one can analyze and optimize the milk production process at a herd level from the breeding and economic point-of-view. In this context, it is important to have a possibility to early predict culling reasons for cows, since, in the case of finding an effective method, it would be possible to modify breeding actions and farm management practices without anticipating the end of the animals’ productive lives. Therefore, the aim of the present study was to verify whether artificial neural networks and a general discriminant analysis may be an effective tool for predicting the culling reasons in cows based on routinely collected first-lactation data. It turned out that they were most effective in predicting culling due to old age and reproductive problems. It is significant because infertility is one of the conditions that are the most difficult to eliminate in dairy herds.

**Abstract:**

The aim of the present study was to verify whether artificial neural networks (ANN) may be an effective tool for predicting the culling reasons in cows based on routinely collected first-lactation records. Data on Holstein-Friesian cows culled in Poland between 2017 and 2018 were used in the present study. A general discriminant analysis (GDA) was applied as a reference method for ANN. Considering all predictive performance measures, ANN were the most effective in predicting the culling of cows due to old age (99.76–99.88% of correctly classified cases). In addition, a very high correct classification rate (99.24–99.98%) was obtained for culling the animals due to reproductive problems. It is significant because infertility is one of the conditions that are the most difficult to eliminate in dairy herds. The correct classification rate for individual culling reasons obtained with GDA (0.00–97.63%) was, in general, lower than that for multilayer perceptrons (MLP). The obtained results indicated that, in order to effectively predict the previously mentioned culling reasons, the following first-lactation parameters should be used: calving age, calving difficulty, and the characteristics of the lactation curve based on Wood’s model parameters.

## 1. Introduction

Routinely collected data on longevity and survivability of cows and their culling reasons may be used for the analysis of dairy cattle management and milk production profitability in individual animals, herds, or populations [1]. Therefore, these data are more frequently monitored in real time and relationships among them are analyzed in the context of animal performance prediction [2].

The studies published so far have indicated multiple possibilities in this regard, starting from a basic analysis of milk production and linear type traits using economic techniques for decision-making [3,4], through the application of survival analysis to the prediction of longevity breeding value in dairy bulls [5,6], the prediction of health problems associated with metabolic diseases in cows [7,8], the analysis of the association between the leptin gene polymorphism and functional longevity of dairy cows [9], the objective evaluation of effective transition cow management at a herd level [10], ending up with the prediction of the first test-day milk yield of dairy heifers [11]. The previously mentioned studies have mostly focused on the analysis of relatively short time periods, i.e., a specific and important moment in cows’ life (e.g., the perinatal period), whereas research on the effective prediction of dairy cow longevity and/or their culling reasons over a longer time span based on routine herd data is still rather scarce. Preliminary studies on culling reasons in cows were carried out by Lacroix et al. [12] and two decades later by Adamczyk et al. [13], who indicated a potential relationship between certain culling reasons and lifetime performance of dairy cows. In addition, Krug et al. [14] developed a model to identify herds with poor welfare based on the Portuguese national cattle database, suggesting, at the same time, the possibility of replacing the laborious and time-consuming procedures required for the Welfare Quality^®^ protocol.

During the cows’ life, the first lactation is one of the most crucial periods. It is associated with the beginning of animal productive life and the change in management conditions, which constitutes a huge challenge for maintaining the optimal welfare level of cows [15,16,17]. This is significant since primiparous cows, as young animals, undergo growth and development, which must be considered in the production and breeding practice [18,19].

In the context of culling decisions, economic and breeding effects of age at first calving and the course of the first lactation are indicated [1]. This results, among others, from an association between the performance of primiparous cows and their lifetime production [20,21]. In this regard, the current attempts at searching for an association between certain production traits and culling reasons in cows are promising [22,23]. However, a qualitatively and quantitatively optimal selection of variables for prediction models and the use of sufficiently accurate analytical methods still remain a challenge.

In the present study, lactation curve parameters estimated from Wood’s model were used as predictors. Wood’s regression curve, as a mathematical equation describing the relationship between milk yield and lactation duration, is frequently used in the studies on the estimation of milk yield in cows [24,25]. This model includes parameters associated with the course of milk yield of a cow during lactation (incline, peak, and decline after the peak).

One of the prediction methods frequently used in animal science is an artificial neural network (ANN). ANN is an information processing system inspired by biological structures such as the human brain. The popularity of ANN results from their ability to reproduce the processes occurring in the brain (incremental information processing, learning new concepts, making decisions, and drawing conclusions based on complex, sometimes irrelevant, or incomplete data), to a limited extent [26]. Therefore, ANN represents a different approach than traditional statistical methods in which it is necessary to define an algorithm and record it in the form of a computer program. Instead, ANN are presented with exemplary tasks and the connections between the network elements and their weight coefficients are modified automatically, according to the assumed training strategy. Besides the ability for self-programming, ANN also show reduced sensitivity to the damages of their structural elements and capability of parallel data processing [27]. There are different types of ANN (feedforward, recurrent, cellular, etc.) among which feedforward ANN consisting of several neuronal layers (an input layer, one or more hidden layers, and an output layer) are very popular. Such ANN are trained in a supervised manner, which means that the desired responses (e.g., culling reasons) are known for each training example (containing cow data). The more recent applications of ANN in animal science include the prediction of milk yield [28], fertility status [29], and assisted or difficult calvings [30] in dairy cows, or the estimation of carcass weight in beef cattle [31], among others. Another, more traditional statistical approach (also belonging to data mining methods) is a general discriminant analysis (GDA). In this method, discriminant function analysis problems are solved using a general multivariate linear model, in which the dependent variables are binary vectors that reflect the class membership of each case (animal) [32,33]. GDA offers more possibilities than traditional discriminant function analysis, based on a classification rule, which allows for the correct classification of cows and the evaluation of classification accuracy depending on the adopted division criteria. In animal science, GDA has been used for dystocia detection in dairy cows [34] or the examination of factors affecting beef tenderness [35], among others.

The following research hypothesis was adopted in the present study. ANN may be an effective tool for predicting culling reasons in cows, based on routinely collected first-lactation data. Moreover, the effects of the prediction made by ANN were compared with those obtained using the GDA.

## 2. Materials and Methods

### 2.1. Animals

The analysis included data on Holstein-Friesian cows (from 466 herds) culled in Poland between 2017 and 2018. The animals were performance recorded. The data were obtained from the SYMLEK Polish National Milk Recording System. SYMLEK is a system of databases (including the results of data analysis for breeding purposes) on the population of dairy cattle under milk recording in Poland. At the breeding level, the system is managed by the Polish Federation of Cattle Breeders and Dairy Farmers, while ZETO Software is responsible for its technical (IT) side.

### 2.2. Data Splitting

Based on the whole dataset of test-day records for the first lactation, the cows were grouped according to the culling reason and the age at first calving. The following culling reasons (R) were analyzed: infectious diseases (R1), respiratory system diseases (R2), low milk yield (R3), nutritive and metabolic diseases (R4), leg diseases (R5), udder diseases (R6), infertility and reproduction problems (R7), old age (R8), accidents (R9), and others (R10). The grouping was carried out in order to plot lactation curves through the calculation of Wood’s model parameters within the categories of culling reasons and age at first calving. Using test-day records, 17 cow groups were distinguished according to the age at first calving (at one-month intervals, from 17 to 34 months of calving, whereas the cows calving at the age of 17 and 18 months were treated as one group). A total of 164 classes (age group × culling reason) were formed in this way. Theoretically, the number of classes should be 170 (17 age groups × 10 culling reasons), but six age groups had missing data for certain culling reasons (Table 1).

### 2.3. Estimation of Wood’s Model Parameters

Based on milk yield from test-day records, the first-lactation curve parameters were estimated separately for each group (age at first calving × culling reason). For this purpose, the mean values of milk yield from test-day records were determined for each lactation stage. Ten lactation stages were distinguished at 30-day intervals (the first lactation stage from 5 to 30 days of lactation, the second lactation stage from 31 to 60 days, the third lactation stage from 61 to 90 days, the fourth lactation stage from 91 to 120 days, the fifth lactation stage from 121 to 150 days, the sixth lactation stage from 151 to 180 days, the seventh lactation stage from 181 to 210 days, the eighth lactation stage from 211 to 240 days, the ninth lactation stage from 241 to 270 days, and the 10^th^ lactation stage from 271 to 305 days). For the description of the lactation curve, the gamma function proposed by Wood [36] was used:

(1) $$y=a\cdot t^{b}\cdot e^{-c\cdot t},$$

where *y* is the milk production (kg) at time *t* (days), *e* is Napier’s constant, *a* is the initial milk yield, *b* is the rate of increase until the peak is reached, and *c* is the rate of decline after peak production.

The regression model parameters were estimated with the quasi-Newton method [37]. A total of 948,010 test-day records (for the first 305-day lactation) from 163,369 cows were used for estimating Wood’s model parameters. The estimated Wood’s model parameters (*a*, *b*, *c*) were used as explanatory (input) variables for further analysis.

### 2.4. Data Editing

When preparing the training set for classification using neural networks, only cows with a complete set of information were used. Records with less than 1 kg of milk, incomplete or erroneous ones (e.g., improbable minimal and maximal values of variables) were removed. In addition, only cows with at least nine test-day records were included in the analysis. The final dataset contained 50,879 cows.

In this dataset, the following explanatory (input) variables were included: X_1_—herd-size (from 3 to 1644 cows), X_2_—age at first calving (from 17 to 34 months), X_3_—lactation length (in days), X_4_—the number of first-lactation test-day records, X_5_–X_7_—Wood’s model parameters (*a*, *b*, *c*, respectively) for individual categories (age at first calving × culling reason). Additionally, the following production traits for the first lactation were used as predictors (minimum, maximum, mean values, and standard deviations, respectively): X_8_–X_11_—daily milk yield (kg), X_12_–X_15_—fat content (%), X_16_–X_19_—protein content (%), X_20_–X_23_—lactose content (%), X_24_–X_27_—dry matter content (%), X_28_–X_31_—urea content (mg/L), and X_32_–X_35_—somatic cell count (thousand/mL). Moreover, first-lactation nominal variables such as X_36_—calving difficulty (according to the scale used for performance recording: easy, spontaneous, difficult, very difficult, abortion, and cesarean section) and X_37_—calving season (spring from 21 March to 20 June, summer from 21 June to 21 September, autumn from 22 September to 22 December, and winter from 23 December to 20 March) were included in the model.

Ultimately, the dataset was randomly divided into a training set (33,071 culling records, 65% of all observations), a validation set (used for controlling the network training process, 7632 records, 15% of all observations) and a test set (used for verifying the predictive performance of the models, 10,176 records, and 20% of all observations). The distribution of continuous and nominal predictors in individual sets is presented in Table 2 and Table 3.

### 2.5. Neural Network Analysis

**Figure 1:**
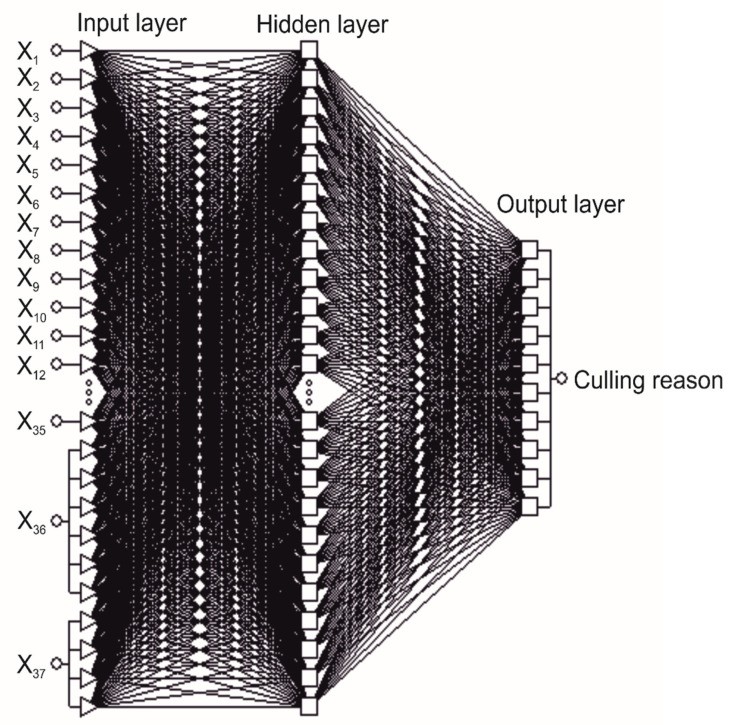
The structure of the multilayer perceptron with one hidden layer and 37 input variables (MLP37).

Different multilayer perceptrons (MLP) with one hidden layer were analyzed. The hidden layer consisted of 5 to 30 neurons (the number of neurons was selected empirically). The number of neurons in the input layer was 45 and the calving season and calving difficulty variables were coded by four and six neurons, respectively (one-of-*n* encoding) (Figure 1). In the input layer, the min-max transformation was used for continuous variables. In the hidden and output neurons, different types of activation functions were verified (linear, logistic, hyperbolic tangent, and exponential). The networks were trained with the Broyden-Fletcher-Goldfarb-Shanno (BFGS) algorithm, which is a powerful second order training algorithm with very fast convergence but high memory requirements due to storing the Hessian matrix [38]. For each analyzed network, a given number of iterations was carried out until reaching the minimum misclassification rate on the validation set. For the evaluation of the network during its training, two error functions were considered, i.e., the sum of squares and cross-entropy. The latter is calculated as the sum of the products of real values and error logarithms for each output neuron.

Based on the obtained results, the classification matrix was created on the test set and predictive performance measures were calculated (the percentage of correctly classified cases from each category and the overall accuracy). In addition, the positive predictive values (PPV) were calculated, which showed the reliability of predictions made by the neural models. Finally, a sensitivity analysis was carried out for ANN, which allowed for the ordering of predictors (input variables) according to their relative importance. This analysis was based on two criteria: an error ratio, i.e., error when input was set to mean divided by the error when input was used (for continuous predictors) or an average error when input was set to all other categorical levels divided by the error when input was used (for categorical predictors), and a rank, which ordered predictors according to their decreasing importance from one (the most important predictor) to 37 (the least important predictor).

### 2.6. Training of the Neural Model with the Most Discriminative Predictors

**Figure 2:**
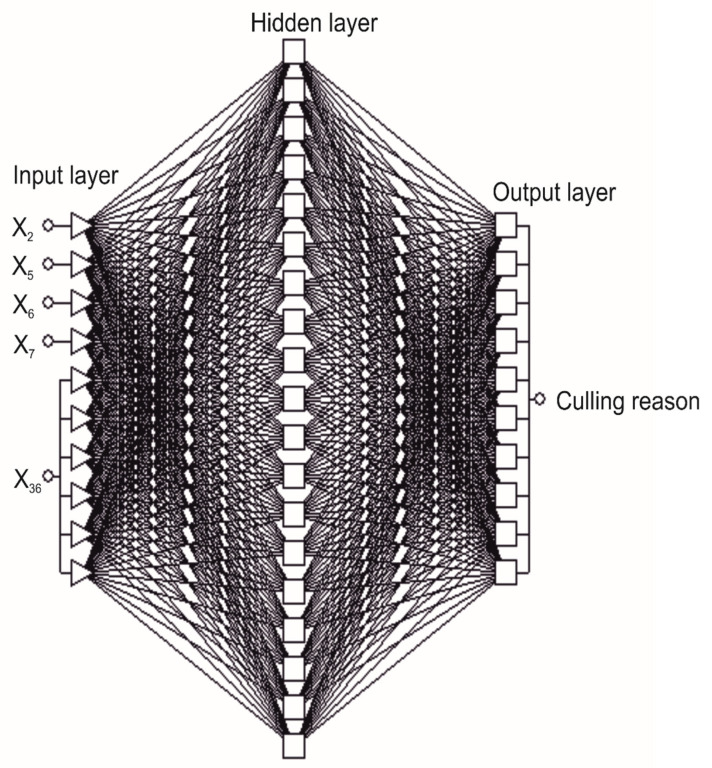
The structure of the multilayer perceptron with one hidden layer and five input variables (MLP5).

Based on the results of sensitivity analysis (Table 4), the set of predictors was limited to the five most discriminative ones, adopting the value of an error ratio above 1.5. The entire procedure was the same as for the networks with the full set of predictors (the selection of the best network out of 10 initial networks, classification matrix, sensitivity analysis, and gains charts). Clearly, there were differences in the number of input neurons (ten, Figure 2).

### 2.7. Discriminant Analysis

Based on the same dataset as for ANN, the GDA was carried out and the classification matrix was created on the test set for the models including all 37 initial or the five most discriminative predictors. The method of the GDA model building was described in more detail by Zaborski et al. [34]. In addition, the predictive performance measures were calculated (the percentage of correctly classified cases from each culling category, the overall accuracy, and PPV).

### 2.8. Gains Charts

In order to better illustrate the predictive abilities of the neural and GDA models, cumulative gains charts were also plotted. These charts show the relationship between the cumulative gains (the proportion of cases from a given culling category among all the cases belonging to this category) and the percentage of cases predicted by the model as belonging to this category in the whole data set [39]. A diagonal crossing the (0,0) and (1,1) points (the baseline) indicates a random model (without any predictive capabilities). Therefore, the curves located above the diagonal are preferred [the closer the line to the (0,1) point, the better the model] [40].

Statistica software (v. 13.3, Tibco Inc., Tulsa, OK, USA) was used for statistical analysis.

## 3. Results

The most effective ANN with 37 predictors had a relatively, highly correct classification rate on the training and validation set (86.73%–96.17% and 87.33%–95.96%, respectively) (Table 5). From among the analyzed ANN, the MLP with one hidden layer and a 45-29-10 structure (the number of neurons in the input, hidden and output layer, respectively) was selected (Figure 1). This perceptron (denoted as MLP37) had the highest correct classification rate on the validation set. The applied training algorithm included 320 iterations. The cross-entropy error function was applied together with the SoftMax activation function in the network output layer. A hyperbolic tangent activation function was used in the hidden layer.

The sensitivity analysis of MLP37 showed that the greatest influence on the output variable was exerted by lactation curve parameters (*a*, *b*, *c*), age at first calving, and calving difficulty. Their error ratio ranged from 8.870 (calving difficulty) to 188.901 (the *a* parameter). Therefore, these variables were used as the only input variables for the network with a reduced set of predictors. The remaining input variables for MLP37 had a much lower error ratio, i.e., below 2 (Table 4).

In comparison with the best networks containing 37 predictors, the networks with a lower number of predictors were characterized by the lower values of a correct classification rate both on the training (71.46%–83.01%) and validation (71.40%–83.52%) set. Among these networks, the MLP with one hidden layer and a 10-19-10 structure (denoted as MLP5) was the most effective (Figure 2). The applied training algorithm included 227 iterations. To evaluate the network performance during its training (like for MLP37), an entropy error function was used, which was applied together with the SoftMax activation function in the network output layer. Similarly, a hyperbolic tangent activation function was used in the hidden layer (Table 5).

As can be seen from Table 3, the most frequent culling reasons in the test set were: reproductive problems (4055 records), udder diseases (1314 records), and accidents and leg diseases (1047 and 1025 records, respectively). Nevertheless, both MLP37 and MLP5 almost always correctly classified culling records from an old age category (R8) (Table 6). A very high correct classification rate (at least 99%) was also found for cows culled due to reproductive problems (R7). In other cases, the percentage of correct classification was 91%–97% for MLP37 (except for low milk yield–R3, for which it was 77%) and 51%–88% for MLP5. On the other hand, the lowest correct classification rate (77% and 55% for MLP37 and MLP5, respectively) was observed for low milk yield (R3). The percentage of correct classification for individual culling reasons obtained with GDA37 and GDA5 was, in general, lower than that for MLP37 and MLP5 (Table 7).

R1—infectious diseases, R2—respiratory system diseases, R3—low milk yield, R4—nutritive and metabolic diseases, R5—leg diseases, R6—udder diseases, R7—infertility and reproduction problems, R8—old age, R9—accidents, and R10—other. The numbers of correctly classified cases are shown on the diagonal.

**Figure 3:**
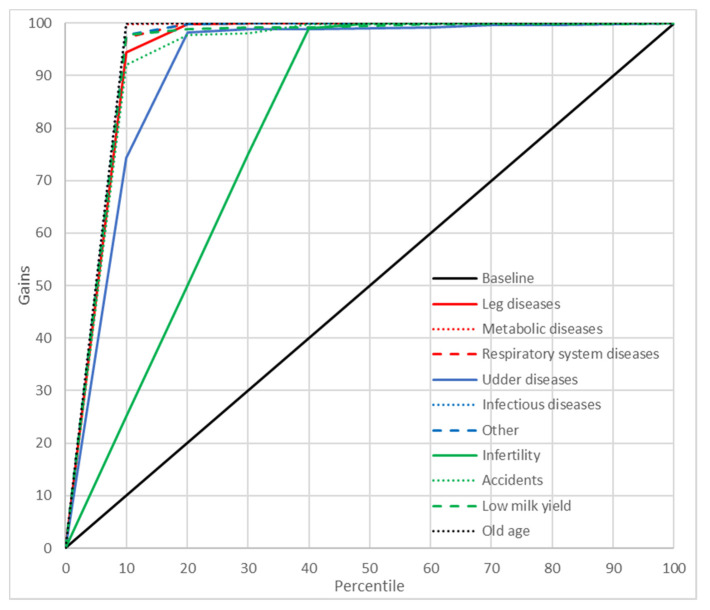
Gain chart for the culling reasons predicted by the multilayer perceptron with one hidden layer and 37 input variables (MLP37). Black, reference line corresponding to a model without discriminatory power.

**Figure 4:**
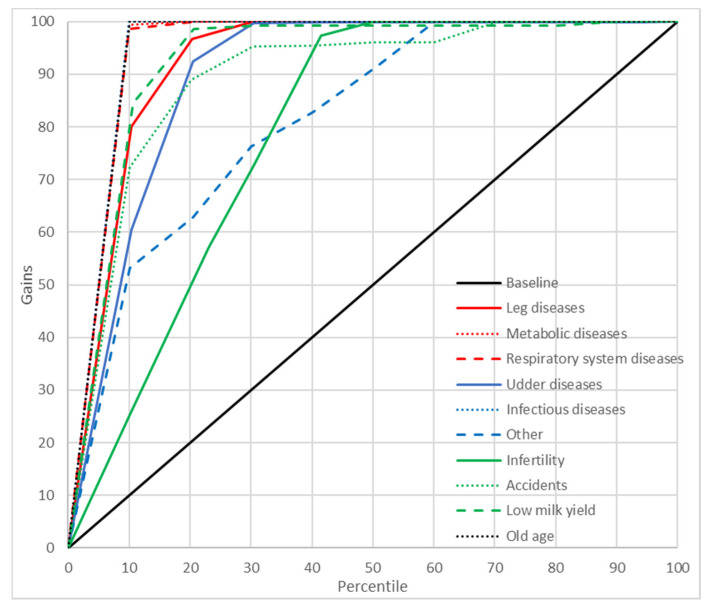
Gains chart for the culling reasons predicted by the multilayer perceptron with one hidden layer and five input variables (MLP5). Black, reference line corresponding to a model without discriminatory power.

**Figure 5:**
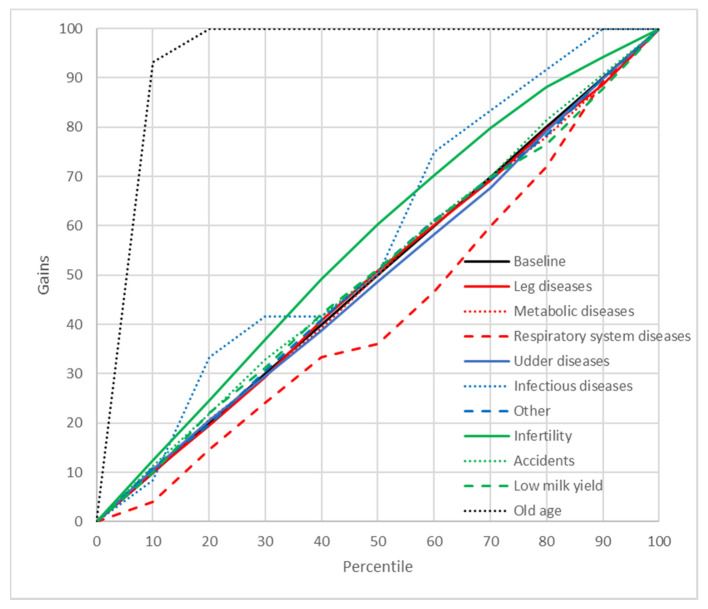
Gains chart for the culling reasons predicted by the general discriminant analysis model with 37 predictors (GDA37). Black, reference line corresponding to a model without discriminatory power.

**Figure 6:**
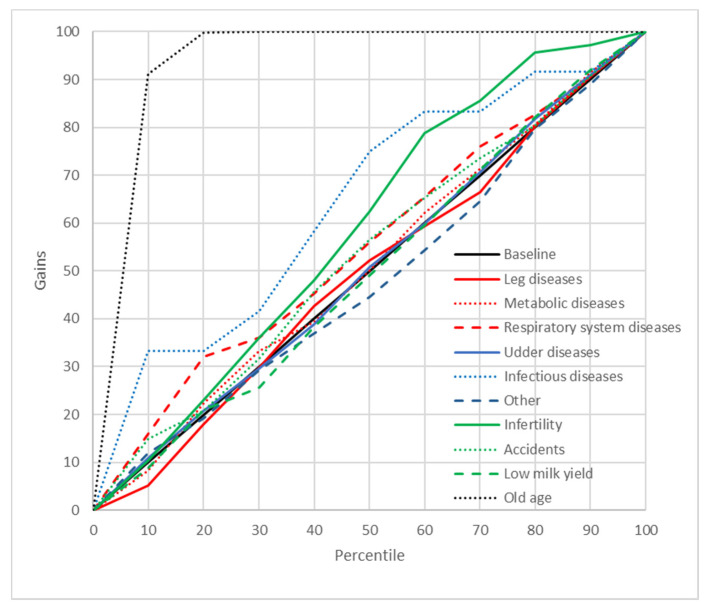
Gains chart for the culling reasons predicted by the general discriminant analysis model with five predictors (GDA5). Black, reference line corresponding to a model without discriminatory power.

A significant indicator of the predictive abilities of ANN and GDA was also the reliability of prediction. In the present study, PPV were used for this purpose (Table 7). In general, these values were quite high for both neural models (88.31–100% for MLP37 and 68.15–100% for MLP5). For GDA, they ranged from 0% to 83.33% (GDA37) and from 0% to 100% (GDA5). In order to get an even better insight into the prediction reliability of ANN and GDA, the cumulative gains charts were plotted and analyzed, which illustrated the relationship between the percentage of correctly classified cases from a given category and the percentage of records from the dataset ordered, according to the predicted probability of the class assignment. It should be emphasized that the gain curves for most culling reasons predicted by ANN were located as much higher than the baseline [near the (0, 100%) point], which indicates a high prediction reliability of the neural models (Figure 3 and Figure 4). However, this time, MLP37 was (like for the previously reported results) more effective than MLP5. When interpreting gains charts for individual categories, one should also consider the percentage of cases from a given category in the whole dataset. Consequently, the course of the curve for udder diseases was not optimal (from the first 13% of observations classified with the highest probability to this category by the model, which about 80% belonged to this class). On the other hand, the curve for reproductive problems passed much closer to the baseline, even though the prediction reliability was very high. This resulted from the fact that the percentage of cases from this category in the whole dataset was about 40%. The gains obtained with both MLP37 and MLP5 were the highest (besides reproductive problems) for such culling reasons as: old age, accidents, or leg diseases. The gains for the GDA models with 37 and five predictors were much lower (Figure 5 and Figure 6). In principle, the gains curves for individual categories (except for old age) were located very close to the baseline, and some curves were even below this line, which shows the uselessness of such models, since better results can be obtained in a purely random manner (without any model).

## 4. Discussion

In the present study, old age was the most accurately predicted culling reason by both MLP37 and MLP5. In addition, prediction reliability for ANN was high for this category, which indicates that almost all animals predicted by ANN to be culled due to old age, really belonged to this category. The gains charts were also nearly optimal. Similar values of individual performance indicators were obtained for GDA. Therefore, in this case, it is really possible to include only lactation curve parameters (from Wood’s model), age at first calving, and calving difficulty as the only predictors in primiparous cows. It may be of great importance from the production practice point-of-view, since, among all culling reasons, old age directly indicates the productive lifespan of dairy cows, considering the longest-living animals. The possibility of an early prediction of the maximum lifespan of cows (based on first-lactation parameters) may provide information required for the assessment of milk production profitability [41] and the modification of breeding programs for dairy cattle in terms of their longevity [42]. It should also be noted that many authors [1,42,43,44] have indicated cow longevity as one of the most important measures of animal welfare. It is highly significant from both a breeding and production point-of-view and due to the increasingly higher sensitivity of dairy product consumers to the human-animal relationship [45]. Therefore, it seems that the prediction of the maximum length of productive life in high-yielding cows should be interesting for both breeders/milk producers and the food industry.

On the other hand, reproductive problems, as the second culling reason (after old age), most accurately predicted by MLP37 and MLP5, belong to the most frequent difficulties encountered in production practice. It is estimated that they account for approximately 20%–40% of culled dairy cows [1,46]. A highly correct classification rate (at least 99% in the case of ANN) for this category was also accompanied by high prediction reliability and high gains (considering the fact that this category was the most frequent one). Cows with reproductive problems were sometimes incorrectly classified to such categories as: leg diseases, udder diseases, accidents, other reasons, and (to a lesser extent) low yield, respiratory system diseases, metabolic diseases, and old age. It may have resulted from the relationship between reproductive problems in cows and other culling reasons. An association between cow fertility and respiratory system diseases [47,48], milk yield level [49,50], metabolic diseases [51,52,53], leg diseases [54,55,56], and udder diseases [57,58] has been shown. In the present study, ANN incorrectly classified reproductive problems in cows in these cases. At the same time, this result supports the suggestion made by Adamczyk et al. [59], who recommended to consider not only the ultimate culling reasons but also the mutual relationships among individual reasons and the life-history of cows when analyzing longevity and indicating culling reasons for these animals. Cows culled due to reproductive problems were also accurately predicted by GDA. However, PPV and gains were lower than those for ANN considering the proportion of this class in the whole dataset. Similar results were obtained for GDA with a reduced set of predictors.

For the potential application of ANN in dairy production, prediction of culling reasons in cows should be considered in a broader context, i.e., concerning the lifetime performance of animals. In this regard, Kumar and Hooda [60] stated that artificial intelligence may be successfully applied to the prediction of lifetime milk yield of cows based on age at first calving, calving interval, and some parameters of the first and second lactation (service period, lactation milk yield, lactation length, and dry period), whereas Bhosale and Singh [61] reported that, for the effective prediction of lifetime milk yield in cross-bred cows with a proportion of Holstein-Friesian genes, it is sufficient to include only the first-lactation parameters in the ANN input layer (lactation length, peak yield, and lactation total milk yield). Moreover, ANN was very effective in this case for both smaller (fewer than 10 cows) and larger herds. Considering the results reported by Bhosale and Singh [61], it should be noted that, in the present study, the predictive abilities of ANN were confirmed based on the first-lactation data, including Wood’s model parameters. Consequently, the effectiveness of ANN in predicting phenotypic milk performance traits is even more important due to the fact that it corresponds to the significant abilities of ANN to predict breeding value of dairy cattle [62].

In this context, a more traditional method such as a discriminant function analysis was much less effective when compared with ANN including 37 or five predictors. In addition, the application of GDA is associated with certain assumptions about predictors, especially multicollinearity, which limits its applicability [33]. Predictors should not be correlated with each other since this causes computational problems. These assumptions, however, are not so important for ANN.

## 5. Conclusions

In the present study, it was shown that artificial neural networks may be an effective method of classifying cows culled due to old age based on routinely collected first-lactation data. Among the remaining culling reasons, a highly correct classification rate was observed for reproductive problems. An association between this culling reason and low milk yield, udder diseases, metabolic diseases, leg diseases, and respiratory system diseases was also confirmed in our study. It should be emphasized that, for the effective prediction of culling reasons, it was sufficient to include such first-lactation traits as calving age, calving difficulty, and the characteristics of the lactation curve (Wood’s model parameters). The confirmed abilities of ANN may constitute a valuable source of information that can be used for breeding programs’ modification in Holstein-Friesian cattle and economic model optimization for dairy herds.

## Figures and Tables

**Table 1:** The number of cows in individual classes based on the age at first calving (AFC) groups and culling reasons (R).

Age Group	AFC	Culling Reason (R)										
		R1 (866)	R2 (2548)	R3 (16,923)	R4 (22,867)	R5 (42,566)	R6 (57,701)	R7 (379,582)	R8 (323,523)	R9 (51,520)	R10 (49,914)	Total (948,010)
1	17–18	0	0	4	4	7	14	38	9	10	6	92
2	19	0	0	8	4	12	17	64	14	11	18	148
3	20	0	2	16	11	23	39	163	22	33	31	340
4	21	3	4	39	55	54	104	359	77	73	68	836
5	22	4	13	95	150	208	320	985	169	227	200	2371
6	23	10	37	204	313	475	674	2046	421	573	480	5233
7	24	12	49	267	443	697	907	2956	639	742	647	7359
8	25	13	49	256	416	698	892	2945	710	772	665	7416
9	26	7	56	226	369	619	758	2529	549	600	614	6327
10	27	10	30	171	282	537	637	2128	382	495	436	5108
11	28	2	29	147	214	405	538	1658	305	401	371	4070
12	29	4	15	98	190	348	462	1345	231	314	297	3304
13	30	2	14	78	129	274	369	1025	182	249	226	2548
14	31	4	17	63	106	201	270	787	152	193	177	1970
15	32	1	11	43	84	160	205	634	103	155	139	1535
16	33	0	5	56	66	131	138	507	82	126	118	1229
17	34	5	8	26	53	104	131	432	73	88	73	993
Total	-	77	339	1797	2889	4953	6475	20,601	4120	5062	4566	50,879

AFC—age at first calving, R—culling reason (R1—infectious diseases, R2—respiratory system diseases, R3—low milk yield, R4—nutritive and metabolic diseases, R5—legs diseases, R6—udder diseases, R7—infertility and reproduction problems, R8—old age, R9—accidents, R10—other). The number of test-day records within each culling reason is given in brackets.

**Table 2:** Mean and standard deviation (SD) of continuous predictors in individual sets.

Variable	Training Set (*n* = 33,071)		Validation Set (*n* = 7632)		Test Set (*n* = 10,176)		Total (*n* = 50,879)	
	Mean	SD	Mean	SD	Mean	SD	Mean	SD
HERD (number of animals)	192.68	290.44	195.97	295.83	191.88	265.77	193.02	291.07
TD	9.54	3.60	9.63	3.62	9.64	3.35	9.87	3.54
AFC (months)	26.28	3.09	26.26	3.06	26.24	3.32	26.27	3.09
DIM (days)	286.86	119.65	287.42	120.27	286.23	110.77	286.81	119.78
*a*	28.56	1.22	28.56	1.21	28.55	1.29	28.56	1.22
*b*	0.13	0.04	0.13	0.04	0.13	0.04	0.13	0.04
*c*	0.06	0.02	0.06	0.02	0.06	0.03	0.06	0.02
MILK (kg)	24.33	6.37	24.34	6.39	24.21	6.00	24.31	6.36
MILKMIN (kg)	17.76	6.63	17.64	6.65	17.65	5.96	17.72	6.61
MILKMAX (kg)	30.48	7.52	30.56	7.60	30.36	7.37	30.47	7.53
MILKSD (kg)	4.52	2.27	4.57	2.34	4.51	2.02	4.52	2.28
FAT (%)	4.12	0.59	4.12	0.59	4.12	0.54	4.12	0.58
FATMIN (%)	3.35	0.62	3.34	0.62	3.36	0.59	3.35	0.62
FATMAX (%)	5.09	1.00	5.09	0.99	5.07	0.97	5.08	0.99
FATSD (%)	0.61	0.36	0.61	0.35	0.60	0.28	0.61	0.35
PROT (%)	3.34	0.29	3.33	0.29	3.33	0.28	3.34	0.29
PROTMIN (%)	2.93	0.26	2.92	0.27	2.93	0.25	2.93	0.27
PROTMAX (%)	3.76	0.47	3.76	0.48	3.76	0.46	3.76	0.47
PROTSD (%)	0.30	0.15	0.30	0.15	0.30	0.14	0.30	0.15
LACT (%)	4.84	0.16	4.84	0.16	4.84	0.15	4.84	0.16
LACTMIN (%)	4.61	0.27	4.61	0.28	4.61	0.26	4.61	0.27
LACTMAX (%)	5.02	0.16	5.02	0.16	5.02	0.15	5.02	0.16
LACTSD (%)	0.14	0.09	0.14	0.09	0.14	0.07	0.14	0.09
UREA (mg/L)	223.64	60.62	222.63	60.79	223.66	60.81	223.49	61.39
UREAMIN (mg/L)	145.70	60.76	144.53	59.66	145.22	58.95	145.43	60.76
UREAMAX (mg/L)	312.24	89.55	310.87	94.24	313.17	90.49	312.22	91.89
UREASD (mg/L)	58.15	28.49	57.95	29.67	58.87	26.27	58.27	29.20
SCC (thousands/mL)	532.26	913.81	528.85	878.79	554.89	738.16	536.27	925.37
SCCMIN (thousands/mL)	89.94	262.47	92.64	250.65	98.53	152.69	92.06	277.86
SCCMAX (thousands/mL)	1836.30	3046.97	1824.38	2969.94	1890.08	2974.92	1845.27	3053.84
SCCSD (thousands/mL)	640.55	1161.20	632.64	1117.94	661.23	999.18	643.50	1160.49
DMSR (%)	13.01	0.73	13.00	0.74	13.00	0.71	13.01	0.73
DMMIN (%)	12.03	0.73	12.01	0.73	12.04	0.71	12.03	0.73
DMMAX (%)	14.14	1.17	14.14	1.16	14.14	1.14	14.14	1.16
DMSD (%)	0.75	0.39	0.75	0.38	0.74	0.33	0.74	0.39

SD—standard deviation, HERD—herd size, TD—number of test-day records, AFC—age at first calving, DIM—days in milk, *a*—initial milk yield (Wood’s model parameter), *b*—rate of increase until the peak is reached (Wood’s model parameter), *c*—rate of decline after peak production (Wood’s model parameter), MILK—average daily milk yield, MILKMIN—minimum daily milk yield, MILKMAX—maximum daily milk yield, MILKSD—standard deviation of daily milk yield, FAT—average fat content, FATMIN—minimum fat content, FATMAX—maximum fat content, FATSD—standard deviation of fat content, PROT—average protein content, PROTMIN—minimum protein content, PROTMAX—maximum protein content, PROTSD—standard deviation of protein content, LACT—average lactose content, LACTMIN—minimum lactose content, LACTMAX—maximum lactose content, LACTSD—standard deviation of lactose content, UREA—average urea content, UREAMIN—minimum urea content, UREAMAX—maximum urea content, UREASD—standard deviation of urea content, SCC—average somatic cell count, SCCMIN—minimum somatic cell count, SCCMAX—maximum somatic cell count, SCCSD—standard deviation of somatic cell count, DM—average dry matter content, DMMIN—minimum dry matter content, DMMAX—maximum dry matter content, and DMSD—standard deviation of dry matter content.

**Table 3:** The number (*n*) and percentage (%) of cows for individual variants of categorical predictors and the output variable in the training, validation, and test set.

Variant	Training Set		Validation Set		Test Set		Total	
	*n*	%	*n*	%	*n*	%	*n*	%
Calving season								
Spring	8593	26.0	1947	25.5	2634	25.9	13,174	25.9
Summer	7608	23.0	1730	22.7	2285	22.5	11,623	22.8
Autumn	8008	24.2	1873	24.5	2466	24.2	12,347	24.3
Winter	8862	26.8	2082	27.3	2791	27.4	13,735	27.0
Calving difficulty								
Unassisted	12,541	37.9	2885	37.8	3835	37.7	19,261	37.9
Easy	18,608	56.3	4293	56.3	5718	56.2	28,619	56.3
Moderate	1491	4.5	355	4.7	468	4.6	2314	4.6
Difficult	148	0.5	35	0.5	53	0.5	236	0.5
Abortions	251	0.8	58	0.8	87	0.9	396	0.8
Caesarean	32	0.1	6	0.1	15	0.2	53	0.1
Culling reason (output variable)								
R1	51	0.2	14	0.2	12	0.1	77	0.2
R2	217	0.7	47	0.6	75	0.7	339	0.7
R3	1131	3.4	303	4.0	363	3.6	1797	3.5
R4	1861	5.6	445	5.8	583	5.7	2889	5.7
R5	3206	9.7	722	9.5	1025	10.1	4953	9.7
R6	4221	12.8	940	12.3	1314	12.9	6475	12.7
R7	13,460	40.7	3086	40.4	4055	39.9	20,601	40.5
R8	2686	8.1	604	7.9	830	8.2	4120	8.1
R9	3283	9.9	732	9.6	1047	10.3	5062	10.0
R10	2955	8.9	739	9.7	872	8.6	4566	9.0

R1—infectious diseases, R2—respiratory system diseases, R3—low milk yield, R4—nutritive and metabolic diseases, R5—leg diseases, R6—udder diseases, R7—infertility and reproduction problems, R8—old age, R9—accidents, and R10—others.

**Table 4:** Sensitivity analysis for the multilayer perceptron with one hidden layer and 37 input variables (MLP37) on the training set.

**Variable**	***a***	***b***	**AFC**	***c***	**CALV**	**DM**	**FAT**	**SEASON**
Ratio	188.901	113.527	45.863	34.940	8.870	1.316	1.284	1.112
Rank	1	2	3	4	5	6	7	8
**Variable**	**LACT**	**SCCSD**	**PROT**	**DIM**	**TD**	**FATMAX**	**LACTSD**	**FATSD**
Ratio	1.112	1.093	1.069	1.056	1.052	1.049	1.049	1.043
Rank	9	10	11	12	13	14	15	16
**Variable**	**SCC**	**MILK**	**UREAMAX**	**LACTMAX**	**PROTMAX**	**MILKMAX**	**DMMAX**	**DMSD**
Ratio	1.041	1.037	1.037	1.036	1.035	1.032	1.030	1.029
Rank	17	18	19	20	21	22	23	24
**Variable**	**PROTMIN**	**FATMIN**	**DMMIN**	**UREAMIN**	**UREASD**	**MILKMIN**	**PROTSD**	**LACTMIN**
Ratio	1.028	1.025	1.024	1.016	1.016	1.015	1.014	1.012
Rank	25	26	27	28	29	30	31	32
**Variable**	**SCCMIN**	**MILKSD**	**SCCMAX**	**HERD**	**UREA**	**-**	**-**	**-**
Ratio	1.012	1.009	1.008	1.008	1.005	-	-	-
Rank	33	34	35	36	37	-	-	-

Ratio—error when input is set to mean divided by error when input is used (for continuous predictors) or average error when input is set to all other categorical levels divided by error when input is used (for categorical predictors), rank—orders predictors according to their decreasing importance from one—the most important predictor to 37—the least important predictor, HERD—herd size, TD—number of test-day records, AFC—age at first calving, DIM—days in milk, *a*—initial milk yield (Wood’s model parameter), *b*—rate of increase until the peak is reached (Wood’s model parameter), *c*—rate of decline after peak production (Wood’s model parameter), MILK—average daily milk yield, MILKMIN—minimum daily milk yield, MILKMAX—maximum daily milk yield, MILKSD—standard deviation of daily milk yield, FAT—average fat content, FATMIN—minimum fat content, FATMAX—maximum fat content, FATSD—standard deviation of fat content, PROT—average protein content, PROTMIN—minimum protein content, PROTMAX—maximum protein content, PROTSD—standard deviation of protein content, LACT—average lactose content, LACTMIN—minimum lactose content, LACTMAX—maximum lactose content, LACTSD—standard deviation of lactose content, UREA—average urea content, UREAMIN—minimum urea content, UREAMAX—maximum urea content, UREASD—standard deviation of urea content, SCC—average somatic cell count, SCCMIN—minimum somatic cell count, SCCMAX—maximum somatic cell count, SCCSD—standard deviation of somatic cell count, DM—average dry matter content, DMMIN—minimum dry matter content, DMMAX—maximum dry matter content, DMSD—standard deviation of dry matter content, CALV—calving difficulty, SEASON—calving season.

**Table 5:** Description of the 10 best multi-layer perceptrons (MLP) with 37 and five predictors.

Ranking	Number of Input Variables	Network Structure	Quality of the MLP [%]		
			Training Set	Validation Set	Test Set
1	37	45-29-10	96.17	95.96	95.94
	5	10-19-10	83.01	83.52	82.99
2	37	45-27-10	90.42	89.70	89.74
	5	10-20-10	79.07	79.42	78.46
3	37	45-29-10	88.70	88.40	88.77
	5	10-6-10	75.95	76.01	75.35
4	37	45-24-10	86.96	86.87	86.56
	5	10-20-10	74.39	74.38	74.14
5	37	45-22-10	86.73	87.33	86.54
	5	10-12-10	71.46	71.40	70.82

**Table 6:** Confusion matrix for the best networks (multilayer perceptrons with one hidden layer and 37 or five input variables) on the test set.

Predicted Culling Reason	No. of Input Variables	Observed Culling Reason									
		R1	R2	R3	R4	R5	R6	R7	R8	R9	R10
R1	37	11	0	0	0	0	1	0	0	0	0
	5	7	0	0	0	0	0	0	0	0	0
R2	37	1	68	2	0	0	0	6	0	0	0
	5	0	49	0	0	0	0	0	0	0	0
R3	37	0	0	279	11	0	0	19	0	0	1
	5	0	0	198	56	0	15	0	0	0	16
R4	37	0	3	50	567	0	0	0	0	0	0
	5	0	0	68	515	0	0	0	2	0	0
R5	37	0	0	1	0	977	2	2	0	18	43
	5	4	15	5	0	755	5	0	0	0	145
R6	37	0	1	18	0	0	1238	0	0	66	2
	5	0	0	33	0	32	996	0	0	362	0
R7	37	0	0	8	4	14	26	4024	1	0	16
	5	0	11	1	0	122	73	4054	0	86	214
R8	37	0	0	0	0	0	0	0	829	0	0
	5	0	0	0	12	0	2	0	828	0	0
R9	37	0	3	4	0	2	26	2	0	961	1
	5	0	0	6	0	5	215	1	0	599	53
R10	37	0	0	1	1	32	21	2	0	2	809
	5	1	0	52	0	111	8	0	0	0	444

R1—infectious diseases, R2—respiratory system diseases, R3—low milk yield, R4—nutritive and metabolic diseases, R5—leg diseases, R6—udder diseases, R7—infertility and reproduction problems, R8—old age, R9—accidents, and R10—other. The numbers of correctly classified cases are shown on the diagonal.

**Table 7:** The correct classification rate for the multilayer perceptrons (MLP) with one hidden layer and the general discriminant analysis (GDA).

Culling Reason	*n*	MLP37				MLP5			GDA37			GDA5		
		Cor.	Incor.	PPV	Cor.		Incor.	PPV	Cor.	Incor.	PPV	Cor.	Incor.	PPV
R1	12	91.67	8.33	91.67	58.33		41.67	100.00	0.00	100.00	0.00	0.00	100.00	0.00
R2	75	90.67	9.33	88.31	65.33		34.67	100.00	6.67	93.33	83.33	1.33	98.67	100.00
R3	363	76.86	23.14	90.00	54.55		45.45	69.47	22.87	77.13	41.91	0.83	99.17	13.04
R4	583	97.26	2.74	91.45	88.34		11.66	88.03	88.16	11.84	70.99	90.05	9.95	66.37
R5	1025	95.32	4.68	93.67	73.66		26.34	81.27	32.20	67.80	46.61	3.22	96.78	76.74
R6	1314	94.22	5.78	93.43	75.80		24.20	69.99	37.98	62.02	47.89	4.19	95.81	20.44
R7	4055	99.24	0.76	98.31	99.98		0.02	88.88	91.39	8.61	58.91	97.63	2.37	49.81
R8	830	99.88	0.12	100.00	99.76		0.24	98.34	90.36	9.64	78.13	91.32	8.68	69.73
R9	1047	91.79	8.21	96.20	57.21		42.79	68.15	2.96	97.04	40.26	0.00	100.00	0.00
R10	872	92.78	7.22	93.20	50.92		49.08	72.08	4.82	95.18	28.00	0.00	100.00	0.00
Total	10,176	95.94	4.06	-	82.99		17.01	-	58.57	41.43	-	52.41	47.59	-

*n*—number of records, MLP37—multilayer perceptron with one hidden layer and 37 input variables, MLP5—multilayer perceptron with one hidden layer and five input variables, GDA37—general discriminant analysis with 37 predictors, GDA5—general discriminant analysis with five predictors, Cor.—correct, Incor.—incorrect, PPV—positive predictive value, R1—infectious diseases, R2—respiratory system diseases, R3—low milk yield, R4—nutritive and metabolic diseases, R5—leg diseases, R6—udder diseases, R7—infertility and reproduction problems, R8—old age, R9—accidents, and R10—others.

## Data Availability

The data are publicly unavailable due to data confidentiality.
